# The chemistry and toxicity of discharge waters from copper mine tailing impoundment in the valley of the Apuseni Mountains in Romania

**DOI:** 10.1007/s11356-017-9782-y

**Published:** 2017-07-25

**Authors:** Piotr Rzymski, Piotr Klimaszyk, Włodzimierz Marszelewski, Dariusz Borowiak, Mirosław Mleczek, Kamil Nowiński, Bożena Pius, Przemysław Niedzielski, Barbara Poniedziałek

**Affiliations:** 1 Department of Environmental Medicine, Poznan University of Medical Sciences, Poznań, Poland; 2 Department of Water Protection, Faculty of Biology, Adam Mickiewicz University, Poznań, Poland; 3 Department of Hydrology and Water Management, Nicolaus Copernicus University, Toruń, Poland; 4 Department of Limnology, University of Gdańsk, Gdańsk, Poland; 5 Department of Chemistry, Poznan University of Life Sciences, Poznań, Poland; 6 Department of Analytical Chemistry, Faculty of Chemistry, Adam Mickiewicz University, Poznań, Poland

**Keywords:** Copper mining, In vitro toxicity, Health risks, Tailings, Elemental composition

## Abstract

Copper mining generates large quantities of waste, tailings, and acid outflows causing long-term environmental impacts and potential threats to human health. Valea Şesei is the largest tailing impoundment in Romania, created by flooding the valley (known as Valea Şesei) of the Metalliferous Mountains (a division of the Apuseni Mountains) with copper mining waste. The present study (i) estimated the total volume of tailings in this area; (ii) screened the concentration of 65 elements (rare earth and platinum group elements, alkali metals and alkali earth metals, transition and post-transition metals and metalloids) and cyanide concentrations in wastewater samples collected from tailing impoundment; (iii) evaluated the toxicity of these water samples using five in vitro bioassays employing human cells isolated from healthy donors and a short-term (1 h) exposure model. The sampled waters were highly acidic (pH 2.1–4.9) and had high electrical conductivity (280–1561 mS cm^−1^). No cyanides were detected in any sample. Water samples collected from the stream (AMD) inflowing to the tailing impoundment were characterized by the greatest concentrations of alkali metals, alkaline earth metals, transition and post-transition metals, metalloids, rare earth elements, and noble metal group. At other sites, the elemental concentrations were lower but remained high enough to pose a relevant risk. The greatest magnitude of in vitro toxic effects was induced by AMD. Observed alterations included redox imbalance in human neutrophils followed by lipid peroxidation and decreased cell survival, significant aggregation of red blood cells, and increased prothrombin time. The study highlights that Valea Şesei is a large sink for toxic elements, posing environmental and health risks, and requiring action to prevent further release of chemicals and to initiate restoration of the area.

**Electronic supplementary material:**

The online version of this article (doi:10.1007/s11356-017-9782-y) contains supplementary material, which is available to authorized users.

## Introduction

Copper (Cu) is one of the most economically important metals with demand expected to significantly increase by 2050 (Elshkaki et al. 2016). Its mining has a significant environmental impact as it is mostly conducted in open pits and generates large quantities of waste. Production of 1 t of Cu typically requires over 150 t of ore to be excavated, crushed, concentrated by froth flotation, and then extracted using different methods depending on the sulfide or non-sulfide nature of the ore (Wills and Napier-Munn 2006). The whole process produces large amounts of tailings, which are the residues of the ore remaining after Cu extraction and contain high levels of toxic metals (e.g., Cd, Fe, Pb, Zn) and metalloids (e.g., As) (Courtney 2013; Wang et al. 2016). Additionally, outflows from mines, known as acid mine drainage, contribute to the allocation of toxic elements dissolved from rocks overrun by water characterized by extremely low pH, primarily due to oxidation of iron sulfide (Akcil and Koldas 2006).

The key issues in decreasing the impact of mining on environmental and human health include (i) mitigation of acid mine drainage effects by preventing oxidation, neutralizing the acid, or collecting the runoff (Akcil and Koldas 2006) and (ii) appropriate management and disposal of tailings (Mleczek et al. 2016). Most conventional impoundment methods involve the construction of surface ponds with raised embankments in which tailings and mine water are systematically deposited downstream, upstream, or centerline (Vick 1990), although methods such as sub-marine disposal or disposal of dewatered tailings as backfill are also in use (Berkun 2005; Fall et al. 2010). Tightened legislation and increased public awareness have forced mine operators to reinforce the stability of embankments and dams, and monitor the process of disposal (Dold 2008). Otherwise, a dam failure and subsequent release of great quantities of waste may cause an environmental disaster and large-scale health threat—a number of such events have already occurred in mining history (Rico et al. 2008).

**Fig. 1:**
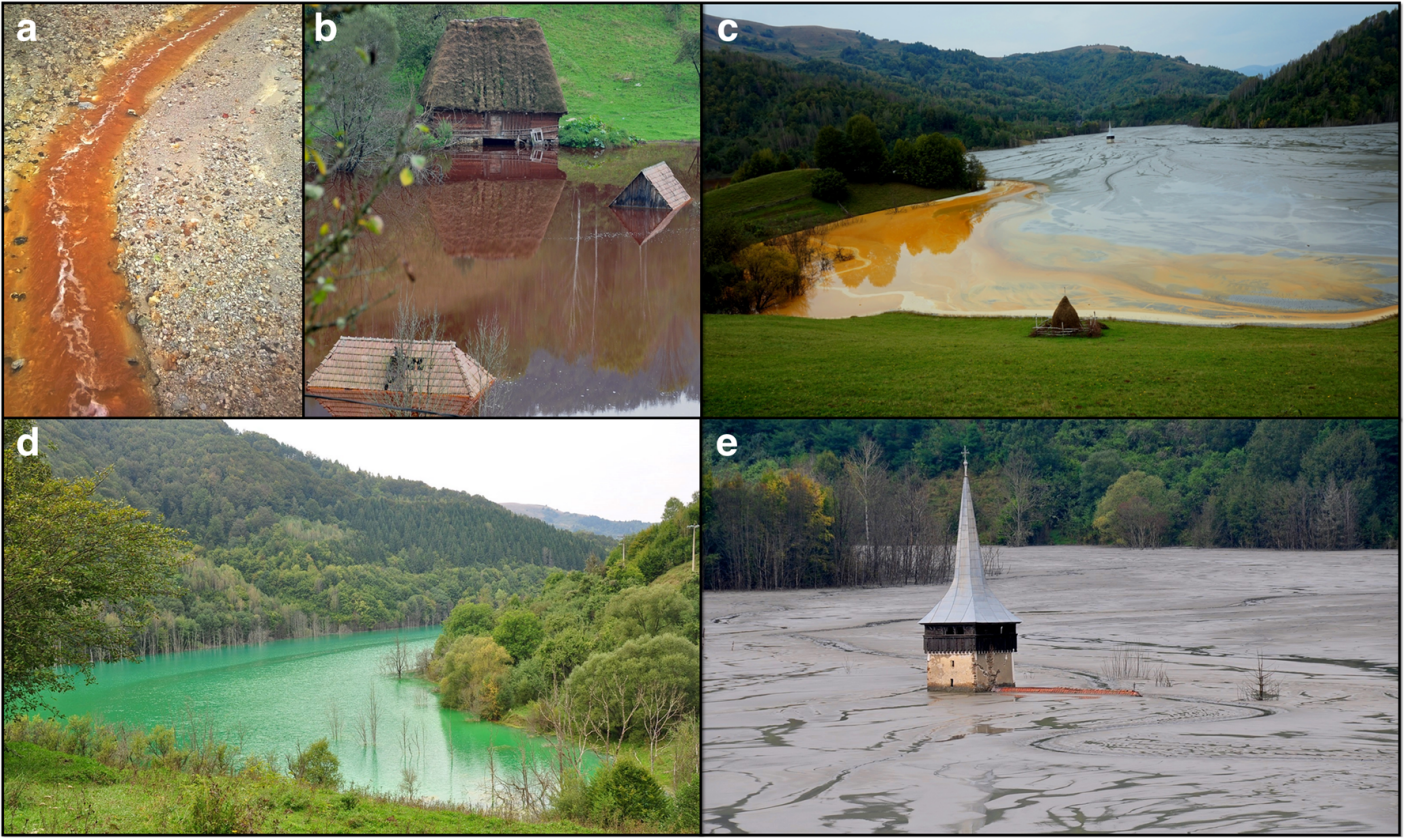
Acid mine drainage inflowing from the Rosia Poieni copper mine to Valea Şesei (**a**) and views of different parts of tailing impoundment (**b**–**e**)

In some of locations, tailings are still deposited on natural surfaces without raised embankments. One of the most striking examples of this is Valea Şesei, the largest tailing impoundment located in the Apuseni Mountains in Romania (Milu et al. 2002). It was created as a consequence of the open pit Rosia Poieni Cu ore exploitation initiated in 1986 and encompasses an area of the former Geamăna village (Milu et al. 2002), now almost entirely engulfed in tailings. Only a few inhabitants still live in the direct vicinity of the impoundment, the rest having been relocated. As the mine operates, the valley is systematically flooded by tailings and mine water while buildings of the former village are literally drowned in waste, from which the tower of its historical church can still be seen partially protruding (Fig. 1).

The assessment of environmental and health risks arising from the presence of such deposits of tailings as found in Valea Şesei is highly important if one considers its size and location, and is likely to require an interdisciplinary approach. Over the years, a number of unverified claims as well as anecdotal evidence regarding threats associated with this area have been raised by the media and various websites. These have included the presence of a high concentration of cyanide compounds and mercury in the wastewaters. As shown by only a few studies, waters draining freely from the ore deposit are contaminated with toxic metals (Milu et al. 2002; Melenti et al. 2011) while the entire area contributes significantly to the pollution of the river Aries and its tributaries (Levei et al. 2013). The wastes are continuously deposited as the mine operates, highlighting the necessity for monitoring of this area as regards toxicological risks.

The present study was undertaken in an effort to evaluate the chemical composition of wastewaters generated by the Rosia Poieni Cu mine and the associated toxicological risks. This was assessed by (i) characterizing the morphometry of the Valea Şesei tailing impoundment; (ii) a broad, multi-element ICP-OES screening (total of 67 elements) and analyses of cyanide concentrations of wastewaters from this area, and (iii) toxicological studies of wastewaters by a battery of in vitro bioassays employing cells isolated from healthy human donors. The present study represents the most up to date information on the chemistry and toxicity of wastewaters from Valea Şesei and introduces biomedical tools as part of an integrated ecotoxicological assessment.

## Materials and methods

### Study area

**Fig. 2:**
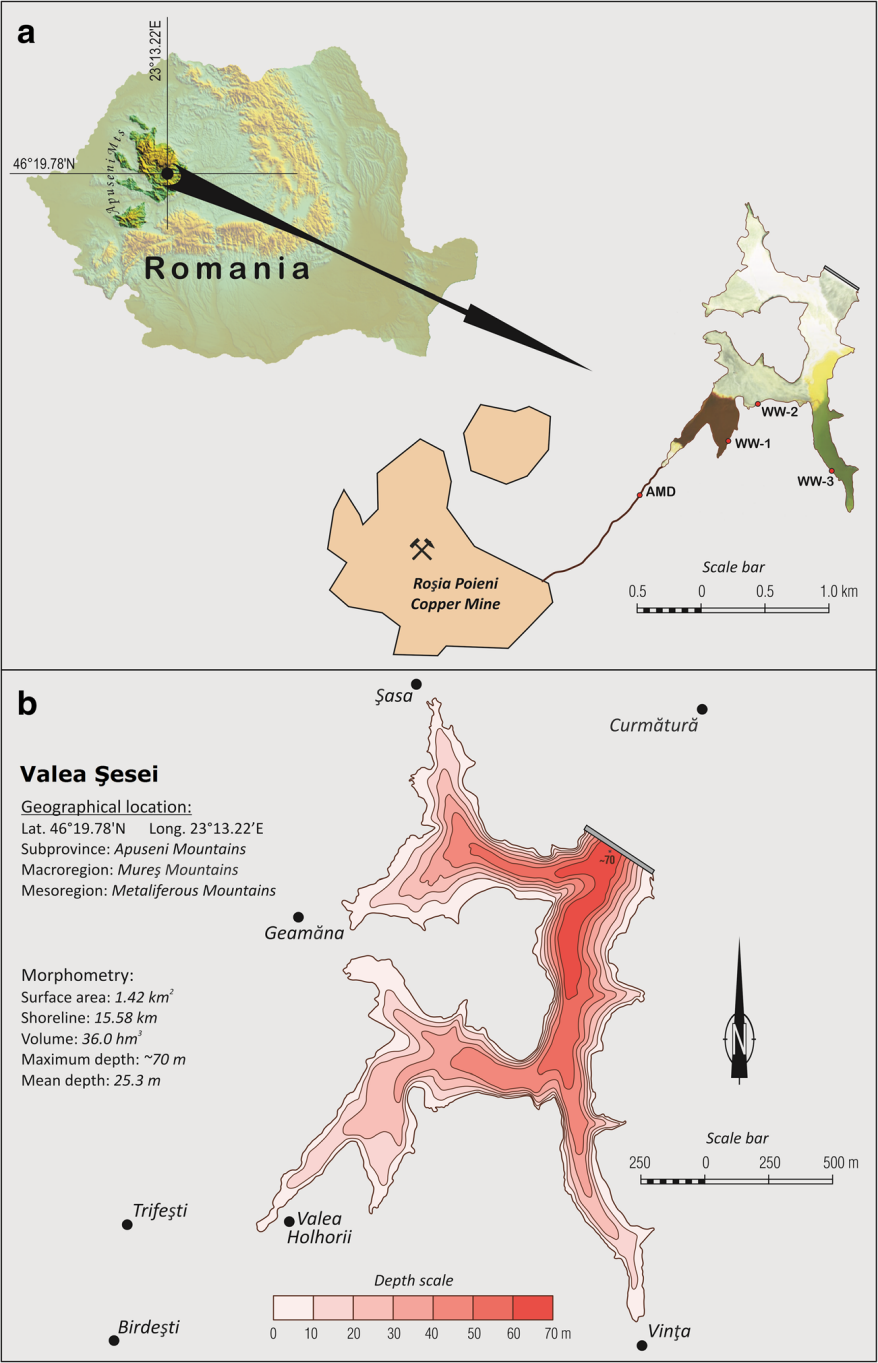
Location of studied area and sampling points (**a**) and morphometric characteristics of Valea Şesei tailing impoundment (**b**)

The Valea Şesei is the largest Romanian tailing deposit located in the Metaliferi Mountains, a division of the Apuseni Mountains (Western Carpathians) created by flooding the dammed valley with tailings and wastewater originating from Rosia Poieni Cu ore, its processing, and smelting (Fig. 2). The exploitation is carried out in the open pit manner and is currently the second largest in Europe, with more than a billion tonnes of porphyry-type ore containing on average 0.36% Cu (Milu et al. 2004). The dam, built from limestone blocks and gravel, is located in the northern part of the impoundment area, has a height of 118 m, and has an inclination of 33° (Duma 1998; Melenti et al. 2011). The nearest water course is the river Aries, located downhill approximately 3.5–4.0 km from the dam, progressing to the rivers Mures, Tisa, and finally through the river Danube into the Black Sea. Geological structures underneath the tailing deposit consist of sedimentary rocks (type not specified) on the west side and crystalline limestone on the east side. It was reported that groundwater up to 8 m below tailing pond are being affected by acid water seepage (Melenti et al. 2011). The surface waters can be found mainly in the southern part where the largest stream inflow is located and in the south-eastern part (Fig. 2a). The area surrounding the tailing storage is hilly and covered by deciduous forests with a prevalence of beech or coniferous trees (Melenti et al. 2011). There is no fence or whatsoever to block an access to impoundment and effluent. The impoundment surroundings are still inhabited by a few locals.

### Quantification of morphometry of tailing impoundment area

An ortophotomap of Valea Şesei originating from 2012 was laid on a military topographic map scale 1:25k (HTM sheets/Hărți topografice militare/: L-34-59-C-d; L-34-71-A-b) using ArcView software (Environmental Systems Research Institute, USA). Due to terrain generalization, the shoreline of the tailing impoundment area frequently crossed contour lines at 690 and 700 m a.s.l. Its course was most consistent with the outline of the 690 m a.s.l. contour line; thus, it was used for cartometric measurements. The topographic map was realized using a 10-m contour interval. The bathymetric map was realized using those contours lower than 690 m that are precise enough to reflect isobaths of the Valea Şesei impoundment area. The total volume of impoundment was evaluated with a bathymetric map applying the formula for the truncated cone (Håkanson 1981).

### Sampling

Sampling took place during summer 2016. The water samples were collected with a telescopic sampler from near the surface of the stream flowing from the area of the Rosia Poieni Cu mine to Valea Şesei (AMD, 46° 19′ 04.3″ N 23° 11′ 55.7″ E) and from three sites WW-1 (46° 19′ 19.6″ N 23° 12′ 33.1″ E), WW-2 (46° 19′ 31.3″ N 23° 12′ 43.8″ E), and WW-3 (46° 19′ 09.8″ N 23° 13′ 22.2″ E) located along the shoreline of the tailing impoundment (Fig. 2). Samples were collected in 0.5-L polypropylene bottles. Three replicates were collected for each site.

### Chemical analyses

#### General physicochemical properties

Electrical conductivity (EC) and pH of collected samples were measured in situ using a portable multi-parameter probe HI9829 (Hanna Instruments, USA).

#### Elemental composition

The water samples were filtered through 0.45-μm filters (Whatman, UK) and acidified with suprapure nitric acid (Sigma-Aldrich, Germany). Elemental analysis was performed using the inductively coupled plasma optical emission spectrometer Agilent 5100 ICP-OES (Agilent, USA). A simultaneous axial and radial view of plasma was obtained by a synchronous vertical dual view (SVDV) using dichroic spectral combiner (DSC) technology. The following common conditions were applied: radio frequency (RF) power 1.2 kW, nebulizer gas flow 0.7 L min^−1^, auxiliary gas flow 1.0 L min^−1^, plasma gas flow 12.0 L min^−1^, charge-coupled device (CCD) temperature −40 °C, viewing height for radial plasma observation 8 mm, accusation time 5 s, three replicates. The calibration was performed using standard analytical solutions (Merck, Germany). A total of 66 elements were analyzed and grouped as follows: alkali metals (Cs, K, Li, Na, Rb), alkaline earth metals (Ba, Be, Ca, Mg, Sr), transition metals (Cd, Co, Cr, Cu, Fe, Hf, Hg, Mn, Mo, Nb, Ni, Ta, Ti, V, W, Zn, Zr), post-transition metals (Al, Bi, Ga, In Pb, Sn, Tl), metalloids (As, B, Ge, Sb, Si, Te), rare earth elements (Ce, Eu, Er, Gd, La, Nd, Pr, Sc, Sm, Dy, Ho, Lu, Tb, Tm, Y, Yb), and noble metals (Ag, Au, Ir, Os, Re, Rh, Ru, Pd, Pt). Specific information on applied wavelengths and detection limits for each element is given in the Supplementary Data (Table S1).

#### Cyanide concentration

After collection, water samples were preserved by adjusting their pH to 10 with sodium hydroxide after which they were centrifuged at 4500*g*. Total cyanide concentration was determined by a continuous flow analysis system with a photometric flow detector SAN++ (Skalar, Netherlands) according to ISO 14403-2: 2012(E). The detection limit was 5.0 μg L^−1^.

### Toxicological studies

#### Experimental design

The toxicological activity of discharge waters collected from Valea Şesei was assessed and compared using a battery of in vitro assays employing human cells. Water samples were prepared by centrifugation at 4500*g* and filtered through the injection filter with a nominal pore size of 0.2 μm (Sartorius, Germany). The potential inducement of oxidative stress and its detrimental effects was tested in isolated neutrophils and included investigations of intracellular reactive oxygen species (ROS) generation, peroxidation of lipids, and cell survival. The potential effect on coagulation parameters, prothrombin time, and international normalized ratio was tested in separated human plasma, whereas red blood cell aggregation rate was assessed in whole blood via erythrocyte sedimentation rate. The final concentration of waters in exposed samples was always 1%. For each assay, peripheral blood samples were collected from three healthy (screened by physical examination, medical history, and initial blood tests) non-smoking and normal weighted (BMI 18.5–24.9) human donors at the Regional Centre of Blood and Blood Treatment in Poznan, Poland, according to accepted safeguard standards and legal requirements.

#### Human neutrophils isolation

Human neutrophils were isolated from blood samples collected in lithium heparin tubes (Becton-Dickinson, USA) using a one-step density-gradient centrifugation on Gradisol G of specific gravity of 1.115 g mL^−1^ (Polfa, Poland) at 400*g* at room temperature for 30 min. The residual erythrocytes were removed from the cell population by hypotonic lysis. The purity of the neutrophils (>90%) was verified by counting under a light microscope after May-Grunwald-Giemsa staining.

#### Intracellular reactive oxygen species assay

Neutrophils were loaded for 30 min at 37 °C in darkness with 20 μM of 2′,7′-dichlorofluorescin diacetate (DCFDA; Abcam, UK), a fluorogenic dye that measures hydroxyl, peroxyl, and other ROS activities within the cell. Neutrophils were then washed, dissolved in PBS, and seeded in a black clear bottom 96-well plate at a density of 20 × 10^4^ cells per well (99 μL aliquots/well). Water samples were added in the amount of 1 μL and the plate was incubated at 37 °C for 1 h. Fluorescence of DCFDA was measured kinetically after 5, 15, 30, and 60 min of incubation using a Synergy HTX multi-mode plate reader (BioTek, USA) at an excitation of 495 nm and emission of 528 nm. The background signal, measured in exposed neutrophils not loaded with DCFDA, was withdrawn from the corresponding samples. The final results were presented as a percentage of a parallel control constituted of neutrophils incubated with 11 μL of PBS.

#### Lipid peroxidation assay

Lipid peroxidation was analyzed using a Lipid Peroxidation Colorimetric/Fluorometric Assay Kit (BioVision, UK) by means of malondialdehyde (MDA) content. Human neutrophils were seeded in a 96-well plate at a density of 20 × 10^4^ neutrophils per well (99 μL aliquots/well) and exposed to 1 μL of each discharge water sample for 1 h at 37 °C. The control was constituted of cells incubated with 11 μL of PBS. After the experiments, cells were harvested from each well and homogenized on ice in 300 μL of provided lysis buffer (with the addition of butylated hydroxytoluene to prevent artificial lipid peroxidation) and centrifuged to remove insoluble material. The resulting 200 μL of supernatants was transferred to a microcentrifuge tube and supplemented with 600 μL of thiobarbituric acid (TBA) to generate an MDA–TBA adduct. To accelerate the reaction, samples were incubated at 95 °C for 60 min and the final product was measured colorimetrically at 532 nm. Three technical replicates were conducted for each donor. The calculated values were compared to a calibration curve prepared using MDA standard (BioVision, UK). The coefficient of variation (*r* ^2^) for the calibration curve was 0.99. The final results were presented as a percentage of a parallel control.

#### Cell viability assay

Cell viability was determined using the colorimetric 3-(4,5-dimethylthiazol-2-yl)-2,5 diphenyltetrazolium bromide (MTT) metabolic activity assay (BioVision, UK) according to the manufacturer’s instructions. Human neutrophils were seeded in a 96-well plate at a density of 20 × 10^4^ neutrophils per well (99 μL aliquots/well) and exposed to 1 μL of each water sample for 1 h at 37 °C. Afterwards, cells were washed, seeded again, and 10 μL of MTT was added to each well for 2 h. Neutrophils were then treated with 10% sodium dodecyl sulfate in 0.01 HCl and incubated for another 6 h in darkness to dissolve formazan crystals. The optical density (OD) of the final product (the formazan crystals) was measured at 570 nm using a Synergy HTX microplate reader (BioTek, USA). The final results were presented as a percentage of a parallel control constituted of neutrophils incubated with 1 μL of PBS.

#### Coagulation assay

The effects of discharge waters on blood coagulation level were assessed with the prothrombin time measurement and calculation of the international normalized ratio (INR). Venous blood was collected in tubes containing 3.2% sodium citrate as an anticoagulant (Sarstedt S-Monovette*®* 9NC). The ratio of blood to anticoagulant was no less than 10:1. Plasma was obtained by centrifugation at 1500×*g* at 21 °C for 15 min, pipetted and divided into five 990 μL aliquots. The first aliquot was used as a control (with 10 μL of PBS), the subsequent aliquots were exposed for 1 h to 10 μL of each water sample at 37 °C. The prothrombin time was measured immediately with the optical nephelometric method using a K-3002 Optic coagulometer (Kselmed, Poland) in a certified hematological laboratory. INR was calculated from the following formula:

$$ INR=\frac{\mathrm{prothrombin}\kern0.5em \mathrm{time}\kern0.5em for\kern0.5em \mathrm{sample}}{\mathrm{prothrombin}\kern0.5em \mathrm{time}\kern0.5em for\kern0.5em \mathrm{laboratory}\kern0.5em \mathrm{reference}\kern0.5em \mathrm{plasma}\kern0.5em } $$

The normative laboratory range for prothrombin time and INR was 12–16 s and 0.9–1.2, respectively. Additionally, the plasma samples (1 mL) were exposed to a 10-fold higher volume of samples (100 μL) to magnify the potential effects; all samples were then photographed after 5 min.

#### Erythrocyte sedimentation rate assay

To assess whether discharge water samples may affect the erythrocyte sedimentation rate (ESR) also known as Biernacki’s reaction, 5 mL of venous blood of healthy donors was collected directly into BD Vacutainer Seditainer™ glass tubes containing 3.2% sodium citrate as an anticoagulant (4:1) in quintuplicate. Water samples were immediately added in the volume of 50 μL; tubes were inverted eight times to mix and inserted into the BD Vacutainer Seditainer™ stand for 5 h. ESR was measured hourly and given as millimeters of sedimented red blood cells.

### Statistical analyses

The results were analyzed using STATISTICA 10.0 software (StatSoft, USA). Differences in elemental composition between the investigated water samples were evaluated using one-way ANOVA with the Tukey post hoc test. In toxicological studies, the paired sample *t* test was applied to compare the difference in effects induced by each water sample with the control. *p* < 0.05 was considered as statistically significant.

## Results

### Morphometric characteristics of tailing impoundment

The detailed morphometry of Valea Şesei tailing impoundment is presented in Fig. 2b. The total surface area is 1.426 km^2^ whereas the volume of deposited tailing amounts to 36.01 hm^3^. The depth is systematically rising in the direction of the dam with mean and maximum depth of 25.3 and 70 m, respectively (Fig. 2b).

### General physicochemical properties

All investigated water samples were characterized by low pH and high electrical conductivity. The determined ranges of pH were as follows: AMD—2.1–2.2, WW-1—2.5–2.6, WW-2—2.8–2.9, and WW-3—4.8–4.9; whereas mean ± SD electrical conductivity was 15,610 ± 80 μS cm^−1^, 7163 ± 57 μS cm^−1^, 3746 ± 62 μS cm^−1^, and 2800 ± 55 μS cm^−1^ for AMD, WW-1, WW-2, and WW-3, respectively.

### Elemental composition

Of the 65 investigated elements, the concentration of 17 of them were below the level of detection (Au, Cs, Er, Ga, Ge, Hf, Hg, Nb, Pb, Pd, Re, Ru, Sb, Sm, Sn, Ta, and Tm).

#### Alkali metals and alkaline earth metals

**Fig. 3:**
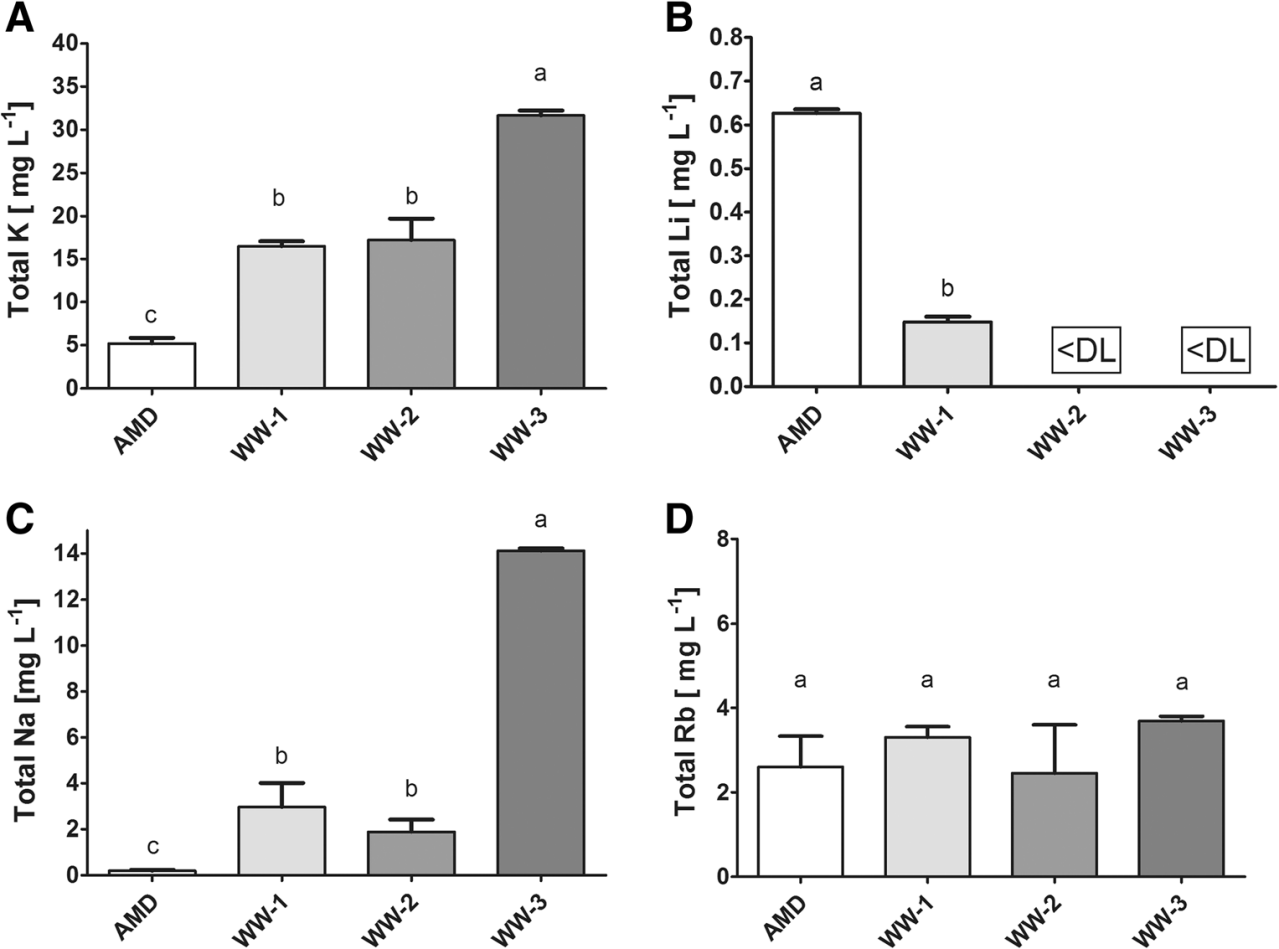
**A**–**D** The concentrations of selected alkali metals in water samples collected at Valea Şesei. Identical superscripts (*a*, *b*, *c*) denote no significant (*p* > 0.05) difference between mean values in rows according to Tukey’s HSD test (ANOVA). *DL* detection limit. Cs was not detected in any sample

The concentration of alkali metals was diverse within the studied waters. The AMD site was characterized by the lowest Na and K concentrations which, in turn, reached the greatest level at WW-3 and exceeded 14 and 30 mg L^−1^, respectively. The highest concentration of Li, exceeding 0.6 mg L^−1^, was found at AMD whereas Rb concentrations were similar at all studied sites and amounted on average to 3.0 mg L^−1^ (Fig. 3).

**Fig. 4:**
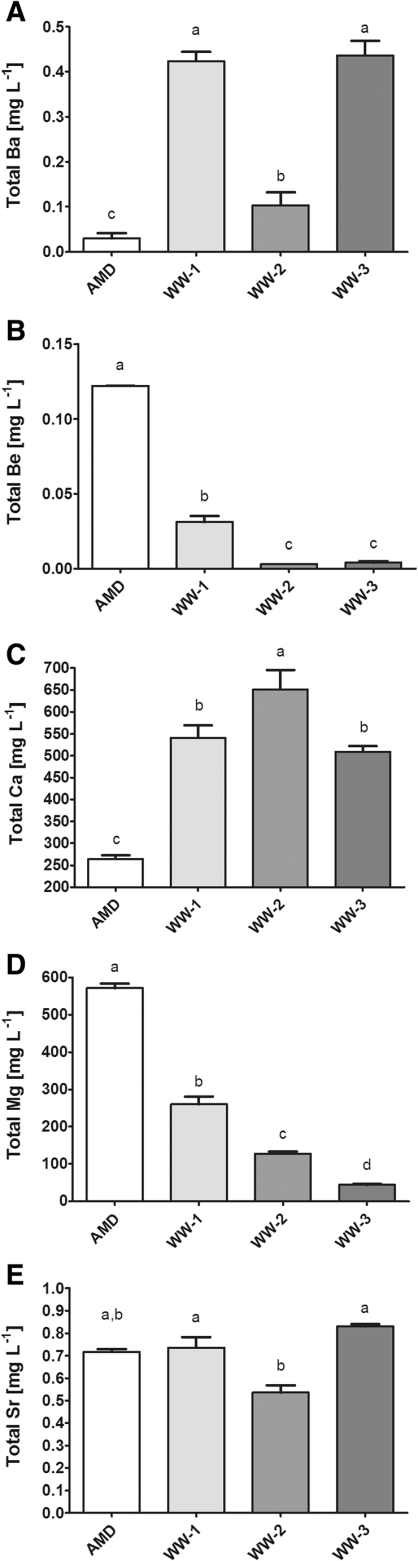
**A**–**E** The concentrations of selected alkaline earth metals in water samples collected at Valea Şesei. Identical superscripts (*a*, *b*, *c*) denote no significant (*p* > 0.05) difference between mean values in rows according to Tukey’s HSD test (ANOVA)

Similarly, the concentrations of alkaline earth metals were also significantly different in the investigated waters. In general, Mg and Ca were the most abundant elements of this group. The AMD site was characterized by the highest concentration of Mg (exceeding 550 mg L^−1^) and Be (over 0.1 mg L^−1^) but decidedly the lowest levels of Ba and Ca. The concentration of Ca was at least 2-fold higher at other sites and amounted to 650 mg L^−1^ at WW-2 (Fig. 4).

#### Selected transition and post-transition metals

Generally, the greatest number of transition metals and their highest concentrations were identified at the AMD site (Table 1). Cr, Ti, and Zr were detected only at this site. Fe, Zn, Cu, and Mn were the most abundant elements of this group, with mean concentrations exceeding 4500, 600, 250, and 60 mg L^−1^, respectively. The AMD site was also characterized by increased levels of Co (over 2.0 mg L^−1^), Cd, and Ni (over 1.0 mg L^−1^ each) and a relatively high concentration of V and W (over 0.5 mg L^−1^ each). The decidedly lowest concentration of transition metals was found at WW-2, with a number of elements below the limit of detection and trace quantities of Fe and Zn (Table 1.)

**Table 1:** Mean ± SD (maximum) content (mg L^−1^) of transition and post-transition metals in water samples (*n* = 3) collected at Valea Sesei

	AMD	WW-1	WW-2	WW-3
Transition metals				
Cd	1.08 ± 0.18^a^ (1.20)	0.22 ± 0.01^b^ (0.24)	<DL	0.01 ± 0.002^c^ (0.0167)
Co	2.20 ± 0.35^a^ (2.47)	0.88 ± 0.07^b^ (0.96)	<DL	0.003 ± 0.006^c^ (0.004)
Cr	0.02 ± 0.02 (0.04)	<DL	<DL	<DL
Cu	259.3 ± 52.4^a^ (291.1)	86.4 ± 6.4^b^ (93.0)	<DL	2.77 ± 0.22^c^ (2.95)
Fe	4669.6 ± 727.9^a^ (5118.2)	410.9 ± 46.7^b^ (459.1)	0.23 ± 0.41^c^ (0.71)	0.006 ± 0.01^c^ (0.01)
Mn	62.3 ± 11.4^a^ (69.8)	25.2 ± 1.8^b^ (26.9)	2.45 ± 0.74^c^ (3.28)	3.82 ± 0.31^c^ (4.09)
Mo	0.01 ± 0.007^a^ (0.015)	0.002 ± 0.003^a^ (0.006)	0.005 ± 0.005^a^ (0.01)	0.02 ± 0.003^a^ (0.025)
Ni	1.48 ± 0.26^a^ (1.65)	0.36 ± 0.03^b^ (0.39)	<DL	0.03 ± 0.007^c^ (0.035)
Ti	0.03 ± 0.03 (0.200)	<DL	<DL	<DL
V	0.508 ± 0.165^a^ (0.606)	<DL	0.001 ± 0.001^b^ (0.003)	<DL
W	0.711 ± 0.228 (0.902)	<DL	<DL	<DL
Zn	610.9 ± 129.9^a^ (692.7)	65.6 ± 7.8^b^ (74.2)	0.04 ± 0.02^d^ (0.06)	4.89 ± 0.35^c^ (5.14)
Zr	0.03 ± 0.03 (0.06)	<DL	<DL	<DL
Post-transition metals				
Al	4488.3 ± 831.1 ^a^ (5014.8)	826.1 ± 68.1^b^ (894.4)	0.19 ± 0.17^d^ (0.34)	8.94 ± 0.14^c^ (9.10)
Bi	0.61 ± 0.10^a^ (0.71)	0.07 ± 0.08^b^ (0.18)	0.14 ± 0.03^b^ (0.17)	0.07 ± 0.03^b^ (0.10)
In	0.50 ± 0.11^a^ (0.62)	0.26 ± 0.05^b^ (0.32)	0.35 ± 0.04^ab^ (0.40)	0.34 ± 0.05^ab^ (0.39)
Tl	0.09 ± 0.09^a^ (0.20)	<DL	0.01 ± 0.02^a^ (0.04)	0.03 ± 0.06^a^ (0.11)

Au, Ga, Hf, Hg, Nb, Pd, Re, Sn, and Ta were not identified in any sample. Identical superscripts (a, b, c, d) denote no significant (*p* > 0.05) difference between mean values in rows according to Tukey’s HSD test (ANOVA)

*DL* detection limit

Al was the most abundant element from the group of post-transition metals. Its high concentrations were identified for each site except WW-2 (Table 1). At AMD, its mean level exceeded 4400 mg L^−1^. The concentrations of other elements of this group were relatively low. The highest concentrations of Bi and In (>0.5 mg L^−1^) were found at the AMD site (Table 1).

#### Metalloids

The highest concentration of detected metalloids was again found at the AMD site. As and Te were identified above the limit of detection only for this site. Compared to WW-1, WW-2, and WW-3, Si concentrations at AMD were 3-fold, 66-fold, and 18-fold higher, respectively (Table 2).

**Table 2:** Mean ± SD (maximum) content (mg L^−1^) of metalloids in water samples (*n* = 3) collected at Valea Sesei

	AMD	WW-1	WW-2	WW-3
As	0.107 ± 0.05 (0.153)	<DL	<DL	<DL
B	6.63 ± 1.38^a^ (7.50)	0.54 ± 0.07^b^ (0.63)	0.05 ± 0.07^b^ (0.06)	0.03 ± 0.02^b^ (0.04)
Si	85.7 ± 14.9^a^ (95.2)	28.1 ± 2.1^b^ (30.2)	1.30 ± 0.07^c^ (1.36)	4.70 ± 0.4^c^ (5.0)
Te	0.51 ± 0.25 (0.75)	<DL	<DL	<DL

Ge and Sb were not identified in any sample. Identical superscripts (a, b, c) denote no significant (*p* > 0.05) difference between mean values in rows according to Tukey’s HSD test (ANOVA)

*<DL* below detection limit

#### Rare earth elements

The investigated waters contained high concentrations of REEs. The total concentration of identified elements ranged from 0.137 (WW-3) to 7.41 (AMD) mg L^−1^. The greatest number of light REEs (Ce, Eu, Gd, La, Nd, Pr, Sc) and heavy REEs (Dy, Ho, Lu, Tb, Y, and Yb) and their highest concentrations were noted for AMD. The general mean concentration of elements at AMD decreased in the following order: Y > Sc > Nd > Gd > Dy > Ce > Pr > Yb > Ho > La > Lu > Eu > Tb. In particular, the concentration of Y at this site was exceptionally high and exceeded 2 mg L^−1^ (Table 3).

**Table 3:** Mean ± SD (maximum) content (mg L^−1^) of light rare earth elements (LREEs) and heavy rare earth elements (HREEs) in water samples (*n* = 3) collected at Valea Sesei

	LREEs							HREEs						Total REEs
	Ce	Eu	Gd	La	Nd	Pr	Sc	Dy	Ho	Lu	Tb	Y	Yb	
AMD	0.65 ± 0.13^a^ (0.74)	0.073 ± 0.01^a^ (0.08)	0.79 ± 0.22 (0.95)	0.24 ± 0.007^a^ (0.252)	0.85 ± 0.18^a^ (0.99)	0.37 ± 0.11^a^ (0.44)	0.95 ± 0.17^a^ (1.06)	0.69 ± 0.12^a^ (0.78)	0.28 ± 0.07^a^ (0.33)	0.10 ± 0.03 (0.12)	0.01 ± 0.01 (0.025)	2.11 ± 0.37^a^ (2.35)	0.30 ± 0.05^a^ (0.34)	7.41
WW-1	0.15 ± 0.01^b^ (0.16)	0.020 ± 0.003^b^ (0.023)	<DL	0.07 ± 0.007^b^ (0.08)	0.20 ± 0.02^b^ (0.21)	0.17 ± 0.01^b^ (0.19)	0.18 ± 0.01^b^ (0.19)	0.13 ± 0.01^b^ (0.14)	0.006 ± 0.006^b^ (0.013)	<DL	<DL	0.56 ± 0.04^b^ (0.60)	0.06 ± 0.006^b^ (0.07)	1.55
WW-2	0.005 ± 0.008^c^ (0.015)	<DL	<DL	0.02 ± 0.04^b^ (0.08)	0.02 ± 0.01^c^ (0.04)	0.14 ± 0.01^b^ (0.15)	<DL	<DL	<DL	<DL	<DL	<DL	<DL	0.185
WW-3	<DL	<DL	<DL	0.02 ± 0.008^b^ (0.028)	0.007 ± 0.01^c^ (0.02)	0.10 ± 0.006^b^ (0.108)	0.004 ± 0.0006^c^ (0.001)	<DL	0.004 ± 0.006^b^ (0.011)	<DL	<DL	<DL	0.002 ± 0.0009^b^ (0.001)	0.137

Er, Sm, and Tm were not identified in any sample. Identical superscripts (a, b, c) denote no significant (*p* > 0.05) difference between mean values in columns according to Tukey’s HSD test (ANOVA)

*DL* detection limit

#### Noble elements

The greatest concentration of noble elements was identified at AMD. Levels of Pt for this sample exceeded 40 mg L^−1^ on average. Relatively high concentrations of Os (over 8 mg L^−1^) and Ir (over 1 mg L^−1^) were also observed. The lowest concentrations of noble metals were detected at WW-2 and WW-3 with Ir and Pt falling below the detection limit (Table 4).

**Table 4:** Mean ± SD (maximum) content (mg L^−1^) of noble metals (NM) in water samples (*n* = 3) collected from area of Valea Sesei in Romania

	NMs					Total NMs
	Ag	Ir	Os	Rh	Pt	
AMD	0.09 ± 0.05^a^ (0.153)	1.93 ± 0.41 (2.28)	8.12 ± 1.65^a^ (9.20)	0.048 ± 0.040^a^ (0.081)	43.01 ± 9.23^a^ (48.7)	53.2
WW-1	0.05 ± 0.03^a^ (0.057)	<DL	0.62 ± 0.06^b^ (0.70)	0.01 ± 0.01^a^ (0.03)	2.65 ± 0.31^b^ (2.98)	3.3
WW-2	0.04 ± 0.02^a^ (0.050)	<DL	0.14 ± 0.01^b^ (0.16)	0.01 ± 0.01^a^ (0.025)	<DL	0.19
WW-3	0.04 ± 0.01^a^ (0.049)	<DL	0.13 ± 0.01^b^ (0.14)	0.005 ± 0.007^a^ (0.0135)	<DL	0.17

Au, Pd, and Ru were not identified in any sample. Identical superscripts (a, b) denote no significant (*p* > 0.05) difference between mean values in rows according to Tukey’s HSD test (ANOVA)

*DL* detection limit

### Cyanide concentration

Total concentration of cyanides was below the limit of detection (5 μg L^−1^) in all investigated samples.

### In vitro toxicity

**Fig. 5:**
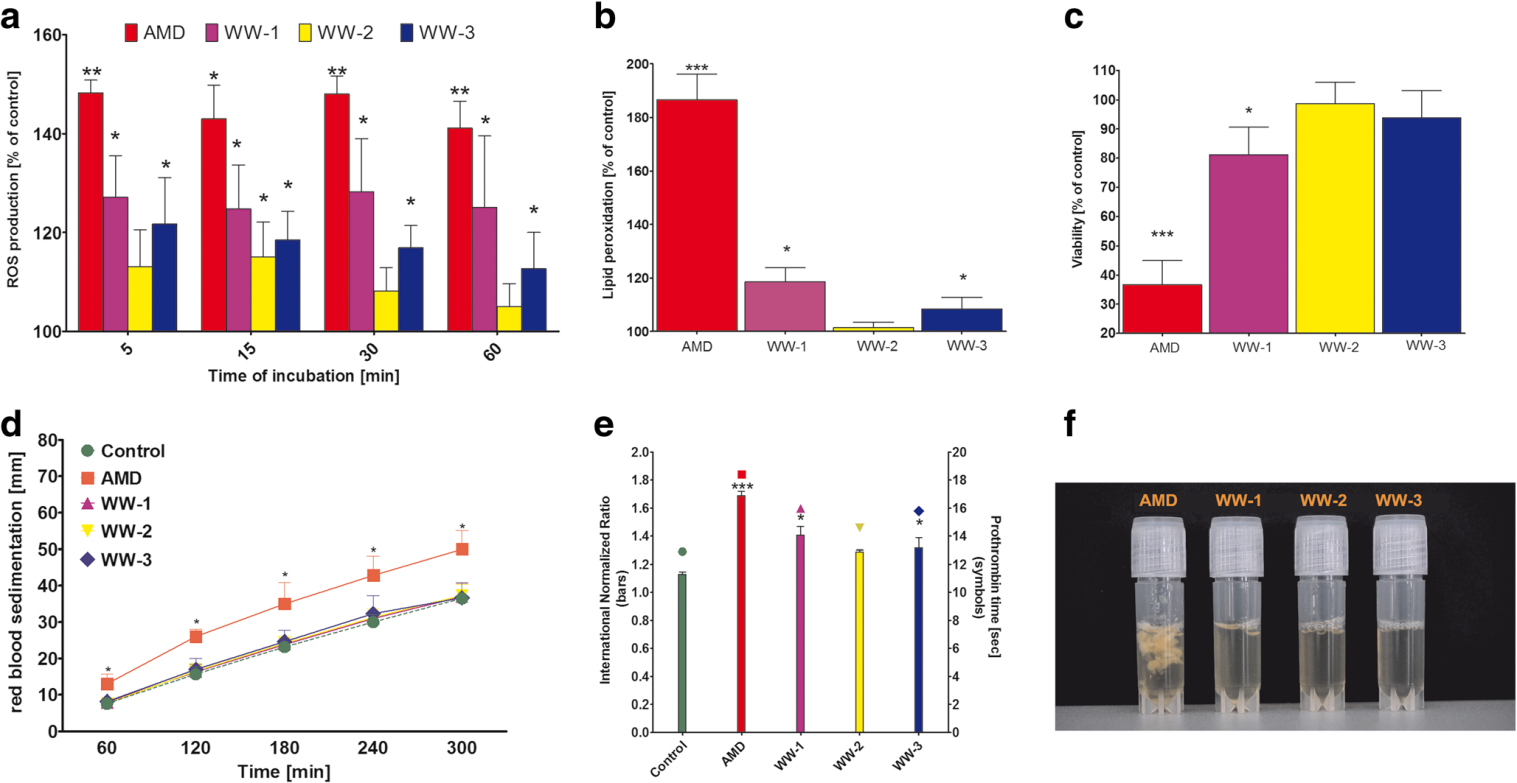
**a** The intracellular ROS concentrations in human neutrophils exposed for 1 h to water samples collected at Valea Şesei measured by means of DCFDA fluorescence and expressed as percentage of control. *Bars* represent mean ± SD from three independent experiments corresponding to different donors. *Asterisks* represent statistically significant difference to the control (*p < 0.05; **p < 0.01; paired sample *t* test). **b** The viability of human neutrophils exposed for 1 h to water samples collected at Valea Şesei measured by means of mitochondrial activity in MTT assay and expressed as percentage of control. *Bars* represent mean ± SD from five independent experiments corresponding to different donors. *Asterisks* represent statistically significant difference to the control (*p < 0.05; **p < 0.01; paired sample *t* test). **c** The erythrocyte sedimentation rate measured for human whole blood exposed for 5 h to water samples collected at Valea Şesei*. Bars* represent mean ± SD from three independent experiments corresponding to different donors. Values recorded for the AMD sample at each interval were significantly different from the corresponding control (*p < 0.05; paired sample *t* test). (**d**) Coagulation parameters measured in human plasma for 1 h to water samples collected at Valea Şesei*. Asterisks* represent statistically significant difference to the control (*p < 0.05; **p < 0.01; paired sample *t* test). **e** Photograph of human plasma (1 mL) exposed for 5 min to water samples (100 μL) collected at Valea Şesei*.* Note white suspension visible in plasma exposed to AMD

Assays employing human neutrophils revealed that exposure to all samples resulted in elevated production of ROS with rapid onset of action. The greatest increase, observed over the entire exposure time, was triggered by AMD and WW-1. Compared to the control, the ROS content after 1 h of exposure was higher by 41.1 and 25.0%, respectively (Fig. 5a). Both samples caused significant lipid peroxidation, as measured by MDA concentrations, elevated by 86.6% for AMD and 18.5% for WW-1 by the end of the experiment (Fig. 5b). This was also followed by greatly decreased cell viability, particularly in the case of AMD. For this sample, neutrophil survival was only of 36.6% of the control level (Fig. 5c).

The addition of 50 μL of the investigated waters to 5 mL of human whole blood samples resulted in significantly increased ESR only under the influence of AMD. This effect was observed after regular 60 min of assay (with 5 mm of mean difference with the control) and remained visible for the next 4 h of incubation. Mean difference in ESR between AMD and the control over the entire experiment was 10.7 mm (Fig. 5d).

Exposure of human plasma to AMD, WW-1, and WW-3 caused prolonged clotting as evidenced by elevated prothrombin time and INR values. In all cases, levels of both INR exceeded the laboratory normative value. The most significant coagulopathic effects were exhibited by AMD—the INR values and prothrombin time amounted to 149.5 and 142.6% of the control level, respectively (Fig. 5e). When the plasma was exposed to a 10-fold higher concentration of each sample (100 μL), formation of white slime could be observed for the AMD-exposed sample in a time as short as 5 min. No visible effects were recorded for other samples (Fig. 5f).

All in all, the tested discharge waters, particularly AMD and WW-1, revealed significant toxicity in human cells causing substantial adverse reactions with short onset time such as inducement of oxidative stress and decreased cell viability in human neutrophils, increased blood plasma prothrombin time and erythrocyte sedimentation rate. The general toxicity of the investigated water decreased in the following order: AMD > WW-1 > WW-3 > WW-2.

## Discussion

The present work is one of the broadest investigations of the multi-element content in wastewaters generated from Cu mining activities. It may thus represent a reference point for observed concentrations in future studies conducted at Valea Şesei or other areas of mining waste discharge.

Although the wastewaters in Valea Şesei were found to possess an extremely enriched chemical matrix as indicated by elemental concentrations and high electrical conductivity, no cyanides were detected. Cyanides, along with Hg compounds, were reported to allegedly occur at high concentrations in Valea Şesei by some media and websites, although cyanides are not employed in Cu ore processing. Instead, separation of Au may employ a very dilute sodium cyanide solution, a method considered to be a much safer alternative to gold extraction by liquid mercury, previously used on the large scale (Adams 2016). In fact, Hg levels were also below the limit of detection in all the samples investigated in the present study. This not only highlights that cyanides and Hg compounds are unlikely to be a subject of concern in Valea Şesei but also that claims of their presence are rather the result of confusing the Cu activities of Rosia Poieni with the historical use of the nearby gold mine Rosia Montanta, now no longer in operation (Stefănescu et al. 2013).

Surprisingly, the investigated wastewaters were characterized by no detectable level of Pb and relatively low levels of As identified only at the AMD site. Both elements are commonly found in mine waters, including acid mine drainage (Stumbea 2013; Migaszewski et al. 2016). However, at very low pH, adsorption of As and Pb on the surface of particles is very efficient and may reduce the concentrations observed in the inflowing water (Zhang 2011). Large quantities of As can also be removed by co-precipitation with Fe(III), the process found to result from microbial As and Fe oxidation (Casiot et al. 2003). Nevertheless, tailings originating from Cu extraction can contain high levels of these elements. As demonstrated by Levei et al. 2013, the tailings deposited in Valea Şesei contained over 30 mg kg^−1^ of Pb and 8 mg kg^−1^ of As but their leachability was low. Immobilization reduces direct environmental risks arising from As and Pb contamination but the problem persists if one considers the total volume, size, and setting of the valley deposits located at Valea Şesei.

The mean contents of detected elements in the investigated samples generally decreased in the following order: Fe > Al > Ca > Mg > Zn > Cu > Si > Mn > Pt > K > Na > Rb > Os > Ir > B > Y > Co > Gd > W > Sr > Ni > Te > Sc > Cd > Dy > In > Li > Ce > Nd > V > Ba > Bi > Pr > As > Ho > Yb > La > Lu > Tl > Ag > Zr > Ti > Cr > Eu > Be > Rh > Mo. Based on the identified contents (particularly for acid mine drainage; AMD site), six main groups of elements could be classified: (i) those that can exceed 1000 mg L^−1^ (Fe and Al); (ii) ranging from 100 to 1000 mg L^−1^ (Ca, Mg, Zn, and Cu); (iii) ranging from 10 to 100 mg L^−1^ (Si, Mn, Pt, and K); (iv) ranging from 1 to 10 mg kg L^−1^ (Na, Rb, Os, Ir, B, Y, and Co); (v) ranging from 0.1 to 1 mg kg L^−1^ (Gd, W, Sr, Ni, Te, Sc, Cd, Dy, In, Li, Ce, Nd, V, Ba, Bi, Pr, As, Ho, Yb, La, and Lu); and (vi) below 0.1 mg L^−1^ (Tl, Ag, Zr, Ti, Cr, Eu, Be, Rh, Tb, and Mo). Their contents were relatively homogeneous as reflected by generally low values of SD calculated for each element. Generally, the concentrations of majority of studied elements largely exceeded those observed in surface waters, including environments under high human pressure (Rzymski et al. 2014; Jitar et al. 2015; Strungaru et al. 2015; Antonowicz et al. 2016; Plavan et al. 2017).

The greatest element concentrations were most decidedly present at the AMD site where mine waters of low pH flow downhill. It is known that acid mine drainage can mobilize and transport large quantities of different elements including toxic metals, metalloids, and REEs (Migaszewski et al. 2014; Migaszewski et al. 2016). In the case of the studied area, waters flowing from the AMD site reach the tailing impoundment and enrich the deposited waste. As shown, the concentration of elements decreased in the order AMD > WW-1 > WW-2 indicating the systematic immobilization of inflowing chemicals. The transfer of dissolved elements from the aqueous phase to the particle phase is due to sorption processes whose efficiency increases with pH (Karlsson et al. 1987). However, it has been demonstrated that various elements are preferably adsorbed within the uppermost layer of the tailings (Müller et al. 2002) and that under certain circumstances, metals and metalloids can be re-released, mostly if extensively oxidized (Moncur et al. 2005). At the WW-3 site, the concentrations were higher than at WW-2 indicating that these waters are probably enriched from other sources (e.g., smaller inflows from the mine area). All in all, the Valea Şesei functions as a large sink for chemical elements (including economically important ones, e.g., Ag, Pt, and other noble metals) inflowing from the mine area and the ore processing plant. There is no doubt that this area must affect the surrounding environment, including the quality of surface and groundwaters, as already evidenced by Milu et al. 2002.

The present study also demonstrated a battery of in vitro bioassays to directly assess the toxicity of collected wastewaters instead of predicting it from elemental concentrations and other physico-chemical parameters. All of these tests employed human blood or isolated human cells, thereby including individual variability and susceptibility. The exposure times were, in turn, short in contrast to those employing cell lines that usually require culturing and a longer assay time to allow for cell responses. For these reasons, the observed effects may be even more relevant for human risk assessment. Despite this, the methods applied in the present work are rarely used to evaluate toxicity of water samples (Zegura et al. 2009), although the employed assays are commonly used in biomedical, pharmacological, and toxicological sciences, including studies on adverse effects of aquatic toxins, e.g., cyanobacterial metabolites (Pizon et al. 2011; Poniedziałek et al. 2014; Poniedziałek et al. 2015b; Al Musawi et al. 2016; Rzymski et al. 2017).

The first set of assays utilized isolated human neutrophils, the most abundant leukocytes, facilitating the innate immune response (Nathan 2006). As previously shown, these cells represent a convenient and rapid in vitro model to test cytotoxicity and elucidate its mechanism of action (Poniedziałek et al. 2015a; Rzymski et al. 2015, 2017). Monitoring of intracellular ROS evaluated whether any of tested wastewaters can induce oxidative stress, an imbalance between ROS generation and biological system ability to readily detoxify the reactive intermediates or to repair the resulting damage (Halliwell and Gutteridge 2007). The potential detrimental outcomes of this phenomenon include DNA damage, protein modifications, lipid peroxidation, and necrotic or apoptotic cell death (Halliwell and Gutteridge 2007; Poniedziałek et al. 2015b; Komosa et al. 2017). In the present study, the two latter effects were also assessed to determine whether any potential change in ROS levels produced adverse effects or whether neutrophils had the ability to cope with them through adaptive responses.

As found, all wastewaters caused an immediate redox imbalance through significant increase in neutrophil ROS levels, particularly high in the presence of AMD and WW-1 samples. This resulted in an increased peroxidation of lipids, a chain reaction initiated by the hydrogen abstraction or addition of an oxygen radical, resulting in the oxidative damage of polyunsaturated fatty acids. If not terminated fast enough, a decrease in membrane fluidity and in the barrier functions of membranes is induced, final products of peroxidation (predominantly MDA and 4-hydroxy-2-nonenal) can induce genotoxicity, and cell death is promoted (Burcham 1998; Ayala et al. 2014). AMD and WW-1 waters reduced cell survival with the viability of neutrophils decreasing by over 60% and nearly 20%, respectively.

While neutrophil-based assays elucidated the mechanism of toxic action of the investigated samples, the present study also aimed to evaluate more general toxic effects. Blood coagulation is an important feature of the vascular system that involves activation, adhesion, and aggregation of platelets along with deposition and maturation of fibrin (Lillicrap et al. 2009), and different chemical compounds are known to interfere with it (Pizon et al. 2011). As demonstrated, all tested samples except WW-2 elevated prothrombin time and INR values above the laboratory normative ranges—the greatest increase was again found for AMD. This implies that exposure of plasma resulted in inhibition of coagulation factors involved in extrinsic and common pathways that triggered impaired fibrinogen clottability (Palta et al. 2014). Increased blood clotting has been shown to play a role in the toxicity exhibited by compounds of various elements, including iron (Rosenmund et al. 1984), Cu (Abou-Shady et al. 1991a), and zinc (Abou-Shady et al. 1991b). In turn, other elements such as cadmium or aluminum are known to cause hypercoagulation (Koçak and Akçil 2006). Therefore, the coagulopathy observed in the present study is most likely to be the result of the simultaneous interaction of many different elements with key players in coagulation pathways—platelets, microparticles, and the clotting factors. It should be stressed that although the differences in prothrombin time and INR values were significant in the present study, the observed effects were not as pronounced as for some chemical compounds, e.g., detergent sclerosants (Parsi et al. 2007).

The last assay employed in the present study was ESR which is routinely used as a non-specific marker of inflammation (Brigden 1998). It is an inexpensive, simple laboratory test conducted on the whole blood (thus in the presence of autologous cellular and serum components that may be physiologically relevant) and allows the results to be easily observable and measured. The ESR depends on the aggregation rate of red blood cells, the phenomenon affected by the size, shape, and number of the red cells, and plasma fibrinogen and globulin levels (Fabry 1987). The present study observed significantly increased ESR only in human blood exposed to AMD. The plausible cause of this includes the destruction of erythrocytes, the subsequent decrease in their count, and eventual increase in sedimentation rate. The association between low hematocrit and high ESR is observed for example in anemia due to altered velocity of the upward flow of plasma resulting in a faster fall of red blood cell aggregates (Brigden 1999). High ESR may also (or additionally) result from a partial loss of albumin (Fabry 1987). Nevertheless, the present study demonstrated that ESR can be a convenient in vitro tool to monitor human-related toxicities for industrially discharged waters.

The magnitude of the observed toxic effects did not directly follow the trends in pH or electrical conductivity of the investigated water samples; it appeared to be associated with their concentrations of elements, particularly toxic metals. The greatest alterations of neutrophil redox balance and cell survival, plasma clotting, and aggregation of red blood cells were induced by AMD, rich in Al, Fe, Cd, As, and REEs. It is most likely that their toxicity was increased due to very low pH. Under such conditions, the bioavailability of various elements is generally increased, e.g., Al occurs as toxic Al^3^+ ion (Krewski et al. 2007). The least adverse effects were revealed by WW-2, still very acidic (pH <3) but generally characterized by the lowest concentrations of elements with evidenced toxicity. The present study demonstrates that the bioassays applied in our study can be an integral part of toxicological evaluation, monitoring, and risk assessment of wastewaters from mining activities.

## Conclusions

The present study demonstrates that wastewaters deposited along with tailings in Valea Şesei in the Apuseni Mountains, Romania, represent a serious environmental and health risk if released on the larger scale. Considering that the mine still operates and generates continuous disposal, there is a strict necessity to prevent any possibility of dam failure and to initiate restoration management in the studied area.

## Electronic supplementary material

ESM 1 (DOCX 20 kb)
