# Supplementary material for: The chemistry and toxicity of discharge waters from copper mine tailing impoundment in the valley of the Apuseni Mountains in Romania
Source: Environ Sci Pollut Res Int. 2017 Jul 25;24(26):21445–58. doi: 10.1007/s11356-017-9782-y (PMC5579155; doi:10.1007/s11356-017-9782-y)
Supplement: Supplementary file 1 — (DOCX 20 kb) [file 11356_2017_9782_MOESM1_ESM.docx]

Table S1. Characteristic of analytical technique of ICP-OES.

|  | **wavelength** | **DL** | **range** |  | **wavelength** | **DL** | **range** |
| --- | --- | --- | --- | --- | --- | --- | --- |
|  | nm | mg L^-1^ | mg L^-1^ |  | nm | mg L^-1^ | mg L^-1^ |
| **Ag** | 328.068 | 0.00086 | DL-20 | **Nb** | 313.078 | 0.00067 | DL-10 |
| **Al** | 396.152 | 0.00053 | DL-20 | **Nd** | 406.108 | 0.0012 | DL-10 |
| **As** | 188.980 | 0.0012 | DL-10 | **Ni** | 231.604 | 0.00092 | DL-20 |
| **Au** | 197.742 | 0.00092 | DL-10 | **Os** | 225.585 | 0.0021 | DL-10 |
| **B** | 249.772 | 0.0047 | DL-20 | **Pb** | 220.353 | 0.0023 | DL-20 |
| **Ba** | 455.403 | 0.00020 | DL-20 | **Pd** | 340.458 | 0.0025 | DL-10 |
| **Be** | 313.042 | 0.0026 | DL-10 | **Pr** | 417.939 | 0.0032 | DL-10 |
| **Bi** | 223.061 | 0.0065 | DL-20 | **Pt** | 203.646 | 0.0021 | DL-10 |
| **Ca** | 422.673 | 0.0036 | DL-200 | **Rb** | 780.026 | 0.0045 | DL-10 |
| **Cd** | 214.439 | 0.00026 | DL-20 | **Re** | 197.248 | 0.0032 | DL-10 |
| **Ce** | 446.021 | 0.0023 | DL-10 | **Rh** | 343.488 | 0.0035 | DL-10 |
| **Co** | 238.892 | 0.00029 | DL-20 | **Ru** | 240.272 | 0.0021 | DL-10 |
| **Cr** | 267.716 | 0.00033 | DL-20 | **Sb** | 206.834 | 0.0012 | DL-10 |
| **Cs** | 697.327 | 0.002 | DL-100 | **Sc** | 361.383 | 0.0024 | DL-10 |
| **Cu** | 327.395 | 0.00027 | DL-20 | **Si** | 288.158 | 0.059 | DL-100 |
| **Dy** | 364.540 | 0.0023 | DL-10 | **Sm** | 442.434 | 0.0026 | DL-10 |
| **Er** | 349.910 | 0.0018 | DL-10 | **Sn** | 283.998 | 0.0067 | DL-10 |
| **Eu** | 420.504 | 0.0034 | DL-10 | **Sr** | 460.733 | 0.00092 | DL-20 |
| **Fe** | 238.204 | 0.00084 | DL-100 | **Ta** | 268.517 | 0.0012 | DL-10 |
| **Ga** | 294.363 | 0.00097 | DL-20 | **Tb** | 350.914 | 0.0027 | DL-10 |
| **Gd** | 342.246 | 0.0034 | DL-10 | **Te** | 214.282 | 0.0011 | DL-10 |
| **Ge** | 209.426 | 0.0039 | DL-10 | **Ti** | 336.122 | 0.0021 | DL-10 |
| **Hf** | 264.141 | 0.0012 | DL-10 | **Tl** | 190.794 | 0.0024 | DL-20 |
| **Hg** | 194.164 | 0.0023 | DL-10 | **Tm** | 336.261 | 0.0031 | DL-10 |
| **Ho** | 348.484 | 0.0031 | DL-10 | **V** | 292.401 | 0.0018 | DL-10 |
| **In** | 230.606 | 0.0025 | DL-20 | **W** | 207.912 | 0.0027 | DL-100 |
| **Ir** | 205.116 | 0.00097 | DL-10 | **Y** | 361.104 | 0.0031 | DL-10 |
| **K** | 766.491 | 0.034 | DL-100 | **Yb** | 328.937 | 0.0014 | DL-10 |
| **La** | 398.852 | 0.00086 | DL-10 | **Zn** | 213.857 | 0.00022 | DL-20 |
| **Li** | 670.783 | 0.00044 | DL-20 | **Zr** | 343.823 | 0.0018 | DL-10 |
| **Lu** | 307.760 | 0.0031 | DL-10 |  |  |  |  |
| **Mg** | 279.553 | 0.00080 | DL-50 |  |  |  |  |
| **Mn** | 257.610 | 0.00021 | DL-20 |  |  |  |  |
| **Mo** | 202.032 | 0.0044 | DL-10 |  |  |  |  |
| **Na** | 588.995 | 0.026 | DL-100 |  |  |  |  |
